# A Localization and Mapping Algorithm Based on Improved LVI-SAM for Vehicles in Field Environments

**DOI:** 10.3390/s23073744

**Published:** 2023-04-04

**Authors:** Lanyi Han, Zhiyong Shi, Huaiguang Wang

**Affiliations:** Department of Vehicle and Electrical Engineering, Army Engineering University of PLA, Shijiazhuang 050003, China

**Keywords:** complex field environment, improved LVI-SAM, pose estimation correction, SLAM

## Abstract

Quickly grasping the surrounding environment’s information and the location of the vehicle is the key to achieving automatic driving. However, accurate and robust localization and mapping are still challenging for field vehicles and robots due to the characteristics of emptiness, terrain changeability, and Global Navigation Satellite System (GNSS)-denied in complex field environments. In this study, an LVI-SAM-based lidar, inertial, and visual fusion using simultaneous localization and mapping (SLAM) algorithm was proposed to solve the problem of localization and mapping for vehicles in such open, bumpy, and Global Positioning System (GPS)-denied field environments. In this method, a joint lidar front end of pose estimation and correction was designed using the Super4PCS, Iterative Closest Point (ICP), and Normal Distributions Transform (NDT) algorithms and their variants. The algorithm can balance localization accuracy and real-time performance by carrying out lower-frequency pose correction based on higher-frequency pose estimation. Experimental results from the complex field environment show that, compared with LVI-SAM, the proposed method can reduce the translational error of localization by about 4.7% and create a three-dimensional point cloud map of the environment in real time, realizing the high-precision and high-robustness localization and mapping of the vehicle in complex field environments.

## 1. Introduction

With the rapid development of science and technology, the automobile industry has become one of the most important industries in the world, playing an essential role in daily life, industrial production, and other fields [1,2,3]. In recent years, autonomous vehicles that can be characterized as driving that do not need a driver but are only navigated by sensors, detection, and computer systems are getting more and more attention and are supposed to bring many benefits to society [4,5]. However, as it remains challenging to make self-driving cars absolutely safe, the public is skeptical that they can be put to mass use anytime soon [6]. Localization and mapping are two important basic capabilities for achieving safe autonomous driving. SLAM technology can quickly map the field environment and effectively assist vehicles in completing automatic driving in complex settings [7,8]. Therefore, it is of great practical significance to carry out research on SLAM technology in complex field environments.

Unlike general outdoor environments, complex field environments have the characteristics of emptiness, terrain changeability, and so on. There are few lidar point cloud features in open environments, so the lidar odometer may fail if utilizing traditional corner points and surface points. Moreover, complex and changeable terrain easily causes bumpiness, generating a great deal of noise in the point cloud image. In addition, the field environment is often an environment where electromagnetic signals are denied, and the GNSS cannot work correctly in such environments. Therefore, it is impossible to use GPS to make regular pose corrections.

SLAM technology can be divided into lidar SLAM and visual SLAM [9,10,11]. Among them, visual SLAM technology is suitable for location recognition, but it is easily affected by low texture, illumination, and intense movement; meanwhile, lidar SLAM technology can capture the details of remote environments with high precision, but are affected by degradation settings [12,13,14,15]. Mur-Artal et al. [16] proposed ORB-SLAM, a classical feature-based visual SLAM system, which operates in real time, in small and large indoor and outdoor environments. Shortly after, Mur-Artal and Tardós [17] presented ORB-SLAM2, a complete SLAM system for monocular, stereo, and RGB-D cameras, including map reuse, loop closing, and re-localization capabilities. Nevertheless, the above two visual SLAM systems are highly affected by environmental conditions and are not suitable for field environments. Gmapping [18,19,20,21], Hector SLAM [22], and Cartographer [23] are the famous two-dimensional (2D) lidar SLAM algorithms but they are also not suitable for field environments due to the small amount of information in single-line lidar. LOAM [24] is a milestone in the 3D lidar SLAM algorithm [25]. However, LOAM only works on smooth surfaces and is sensitive to unstructured features, such as bumps and leaves in outdoor environments.

Multi-sensor fusion SLAM technology combines a variety of sensors to make up for the shortcomings of each sensor and further improve the robustness and accuracy of the SLAM system [26,27,28,29]. It has therefore become a hot topic in SLAM research in recent years. Qin et al. [30] proposed VINS-Mono, a well-known visual inertial odometry. In this method, the pre-integral constraints of the Inertial Measurement unit (IMU) between keyframes and the observation constraints of visual feature points are used to construct the factor graph optimization problem, and loop constraints are added to back-end optimization. However, as the tracking trajectory becomes longer, loop detection and graph optimization become more and more time-consuming, which will have a high impact on the real-time performance of the algorithm. V-LOAM, which was presented by Zhang and Singh [31], is a high-precision visual and lidar fusion SLAM system based on LOAM, but the system cannot eliminate the continuous pose estimation error due to the lack of loop closure. Shan et al. [32] introduced LIO-SAM, which is also based on the LOAM framework; it uses IMU pre-integration to remove the distortion of the lidar point cloud and provides initial values for the registration of the lidar point cloud. The algorithm can also choose to periodically add GPS information to correct the pose and eliminate the cumulative drift to realize long-term localization and navigation. Nevertheless, the algorithm relies on GPS signals to eliminate cumulative drift, so it is not suitable for the GNSS-denied field environments. Shan et al. [33] then proposed LVI-SAM, a SLAM method for fusing lidar, vision, and IMU based on factor graph optimization. The method is composed of two subsystems tightly coupled, among which, the visual-inertial subsystem is based on the VINS-Mono algorithm, and the lidar-inertial subsystem is based on the LIO-SAM algorithm. The algorithm has good experimental results in the data set collected by walking, but when the speed is increased and the road surface is bumpy, the localization accuracy and the map quality will decline.

To solve the above problems, this paper proposed a state-of-the-art lidar, camera, and inertial measurement unit fusion SLAM system to achieve accurate and robust localization and mapping in field environments. The algorithm improves the corner and planar point-based front end of lidar SLAM in LVI-SAM, and the lidar odometry consists of two nodes: the pose estimation node and the pose correction node. In the pose estimation node, the method of point cloud registration from coarse to fine is adopted. The Super4PCS [34] algorithm is used for point cloud coarse registration to provide an initial estimation for subsequent fine registration of the point cloud; finally, the ICP [35,36] algorithm is used for interframe pose estimation in the point cloud fine registration stage. Due to the errors that inevitably occur in interframe pose estimations, a pose correction node was designed. In this node, the NDT [37] algorithm based on dynamic voxel storage is utilized to periodically correct the localization results at a frequency lower than that of the pose estimation node. By combining the above two nodes, both the accuracy of the pose estimation of the lidar odometry and the real-time performance of the pose estimation are guaranteed.

The remainder of this paper is organized as follows. Section 2 introduces the proposed multi-sensor fusion SLAM framework, including the overall architecture, the pose estimation node, and the pose correction node. Then, the quality of the experiment is evaluated in Section 3. Finally, the work of this paper is summarized in Section 4.

## 2. Materials and Methods

### 2.1. Problem Formulation

In the multi-sensor fusion SLAM algorithms, the lidar odometry is an essential part of the SLAM system performance. The lidar odometry solves the problem of point cloud registration and pose estimation. Assume that the point clouds *P* and *Q* are source point clouds and target point clouds in two consecutive point cloud frames, respectively. The goal of the point cloud registration is to find the most appropriate interframe pose R and t of the point clouds to minimize the error between the estimated pose and the ground truth value. In this study, the main goal is to solve the above problem in the unstructured, bumpy, and GNSS-denied field environments.

### 2.2. Brief Review of the LVI-SAM Algorithm

The LVI-SAM algorithm can be divided into two subsystems: a lidar-inertial subsystem and a visual-inertial subsystem. Based on the LIO-SAM algorithm, the lidar-inertial subsystem uses the lidar point cloud and IMU data to calculate the incremental IMU and lidar pose estimation after data preprocessing. The subsystem optimizes the local pose in the front-end factor graph and adds the global loop closure constraint and map matching constraint in the back-end factor graph. After nonlinear optimization, the global pose and the corresponding global map can be obtained. Based on the VINS-Mono algorithm, the visual-inertial subsystem utilizes the monocular visual images and IMU measurement. It completes the system initialization and local pose estimation by tracking the Lucas–Kanade (LK) optical flow and IMU pre-integration. The back end uses the pose graph optimization and sliding window framework to construct the joint visual-inertial residual function and get the global pose estimation.

**Figure 1:**
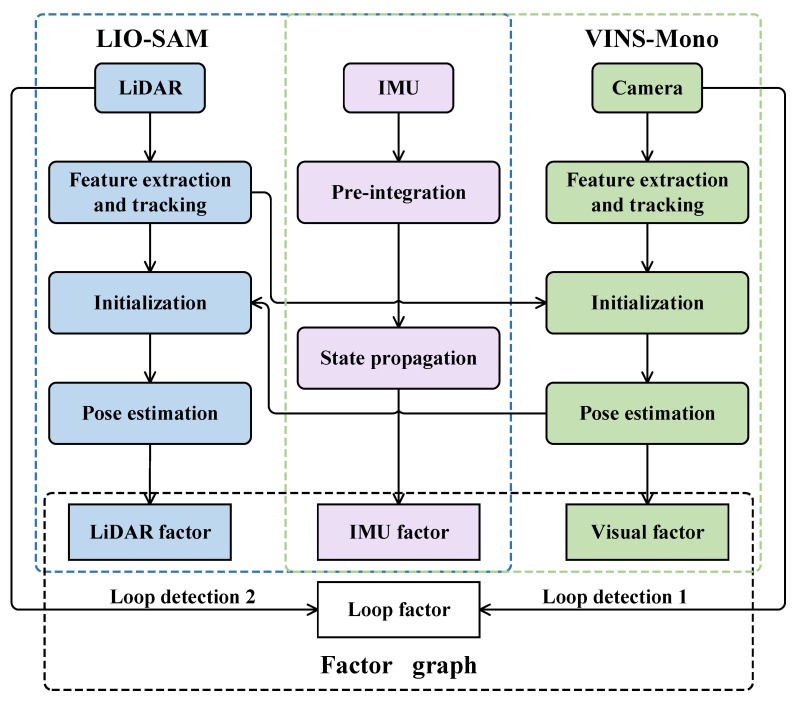
The principle of the LVI-SAM algorithm.

In addition, the two subsystems are tightly coupled. The visual-inertial subsystem utilizes the estimation of the lidar-inertial subsystem to complete the initialization, and the measurement of the lidar provides the depth prediction for the vision. Meanwhile, the lidar-inertial subsystem uses the estimation of the visual-inertial subsystem as the initial value to complete the frame matching. Moreover, the LVI-SAM can still work when the lidar-inertial or the visual-inertial subsystem fails. A secondary loop detection method is designed, significantly improving the robustness of the system in scenes lacking texture or features. Figure 1 shows the principle of the LVI-SAM algorithm.

### 2.3. Overview of the Proposed Lidar Odometry

**Figure 2:**
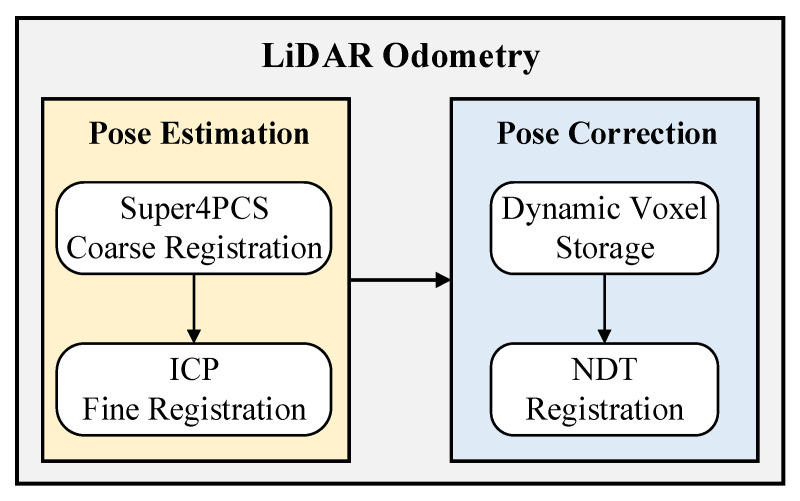
Schematic diagram of the improved lidar odometry.

According to the complex characteristics of field environments, the lidar front end of LVI-SAM was improved. First, there are few lidar feature points in open field environments. The lidar odometry may fail if traditional corner points and planar points are used. Second, the terrain in the field environment is changeable and prone to bumps, resulting in a significant amount of noise in the point cloud image. To solve the above problems, the corner and planar point-based front-end of lidar SLAM in LVI-SAM is improved in this paper. The lidar odometry is composed of two nodes: the pose estimation node and the pose correction node. A schematic diagram of the improved lidar odometry is shown in Figure 2.

### 2.4. Design of the Pose Estimation Node

In the pose estimation node, point cloud registration moving from coarse to fine is adopted. The Super4PCS algorithm is used for point cloud coarse registration to provide initial estimations for subsequent point cloud fine registration, and the ICP algorithm is used for interframe pose estimation during the point cloud fine registration stage. The specific algorithm flow for the pose estimation node is as Algorithm 1.

The theoretical core of the Super4PCS algorithm is that the relative position of the intersection of two-line segments remains unchanged under affine transformation. The affine invariant can be expressed as: in the plane, the point set {*a*, *b*, *c*, *d*} comprises four coplanar and non-collinear points. There must be an intersection point *e*, and its relative position with points in {*a*, *b*, *c*, *d*} can be expressed as:

(1) $$\left\{\begin{array}{c} r_{1}=\left\|a-e\right\|/\left\|a-b\right\|\\ r_{2}=\left\|c-e\right\|/\left\|c-d\right\| \end{array}\right..$$

**Figure 3:**
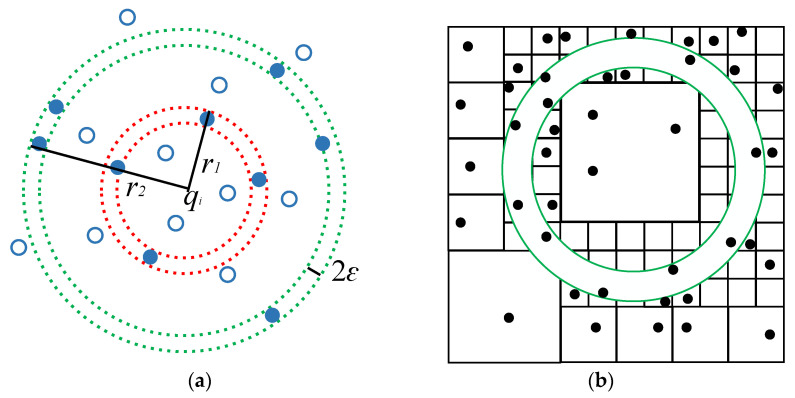
Sphere extraction model of the Super4PCS algorithm. (**a**) Point base set extraction. (**b**) Two-dimensional subdivision unit.

Assume that the point clouds *P*, *Q* are source point clouds and target point clouds, respectively. First, four coplanar and non-collinear points (*p*_1_, *p*_2_, *p*_3_, *p*_4_) in the point cloud *P* are selected as the point base, and the distance between the points is made as large as possible. Then, in the point cloud *Q*, *q_i_* (*i* = 1, 2, …, *n*) is taken as the center of the sphere and *r* as the radius to make the sphere. Figure 3a shows the two-dimensional expression. *n* spheres can be made in *Q* to form a set of spheres. Given the error limit *ε*, all points in the interval [*r* − *ε*, *r* + *ε*] meet the requirements, as shown by the blue points in Figure 3a. The point cloud *Q* is continuously subdivided to store the points in the interval [*r* − *ε*, *r* + *ε*], as shown in Figure 3b. The blue points in the figure are the extracted point pairs, while the black points are the points that do not meet the requirements.

After extracting the point pairs, the affine invariant is used to extract (*q*_1_, *q*_2_, *q*_3_, *q*_4_) from *Q* corresponding to (*p*_1_, *p*_2_, *p*_3_, *p*_4_). Let (*p*_1_, *p*_2_) be *r*_1_ and (*p*_3_, *p*_4_) be *r*_2_; then, the constraint condition is:

(2) $$\left\{\begin{array}{c} e_{1}=q_{1}+r_{1}(q_{2}-q_{1})\\ e_{2}=q_{1}+r_{2}(q_{2}-q_{1}) \end{array}\right..$$

**Figure 4:**
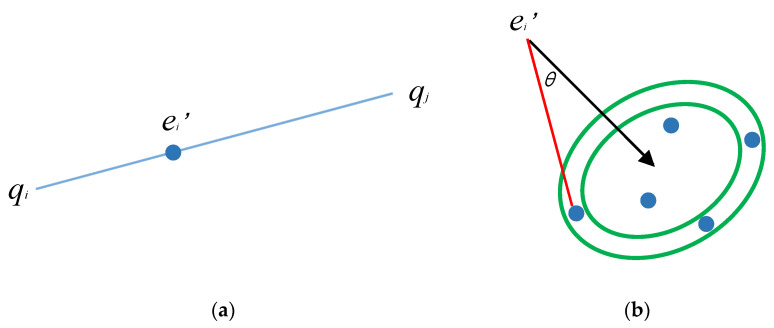
Point base extraction model of the Super4PCS algorithm. (**a**) The affine invariant property. (**b**) Correct point base extraction.

*S*_1_ is defined as the set of point pairs in *Q* corresponding to *r*_1,_ and *S*_2_ as the set of point pairs in *Q* corresponding to *r*_2_. Then, the corresponding direction vector for any pair of points in *S*_1_ and *S*_2_ is calculated and stored. The intersection point *e* can be obtained by the affine invariant property, as shown in Figure 4a. The interval [*r* – *ε*, *r* + *ε*] is given according to the direction vector and angle *θ*, so that four single points can be obtained in *Q*, as shown in Figure 4b. After completing the matching of point pairs, the registration is carried out.

After coarse registration with the Super4PCS algorithm, a good initial value is provided for the ICP algorithm, and then fine registration with the ICP algorithm is performed. Due to the large amount of point cloud data, direct fine registration with the ICP algorithm would be time-consuming, so the point cloud is first filtered by the voxel grid. The point cloud is divided into several grids of the same size and the point cloud data in each grid are extracted with the center of gravity. The center of gravity is used to replace all the point clouds in the grid to achieve the down-sampling of the point cloud data. Taking one of the grids as an example, assume that there are *m* points in the grid. Then, the center of gravity can be calculated using Formula (3):

(3) $$\left(x,y,z\right)=\left(\frac{1}{m}\sum_{i=1}^{m}x_{i},\frac{1}{m}\sum_{i=1}^{m}y_{i},\frac{1}{m}\sum_{i=1}^{m}z_{i}\right).$$

*R_i_* is defined as the rotation matrix and *t_i_* as the translation matrix calculated by the Super4PCS algorithm. Then, pose transformation for the points in the point cloud *Q* is performed:

(4) $$Q^{\prime }=R_{i}Q+t_{i}.$$

The least-squares method is used to minimize the Euler distance between all matches, and the loss function is calculated using Formula (5) and iterating (*R*_1_, *t*_1_) continuously until the algorithm is convergent:

(5) $$E(R_{1},t_{1})=\frac{1}{n}\sum_{i=1}^{n}{\left\|p_{i}-(R_{1}q_{i}+t_{1})\right\|}^{2}.$$

**Algorithm 1:** From coarse to fine pose estimation algorithm.**Input:** Point cloud *P*, *Q*, Error limit *ε*.**Output:** Pose estimation result *R*_1_, *t*_1_.1. Select four coplanar and non-collinear points (*p*_1_, *p*_2_, *p*_3_, *p*_4_) in the point cloud *P* as the point base;2. Take *q_i_* (*i* = 1, 2, …, *n*) as the center of the sphere and *r* as the radius to make *n* spheres in the point cloud *Q*;3. Store the points in the interval [*r* − *ε*, *r* + *ε*] as the extracted point pairs;4. Extract (*q*_1_, *q*_2_, *q*_3_, *q*_4_) from *Q* corresponding to (*p*_1_, *p*_2_, *p*_3_, *p*_4_) according to Equation (2);5. Carry out the coarse registration to get an initial pose estimation *R_i_*, *t_i_*;6. Divide the point cloud into several grids of the same size and extract the point cloud data in each grid with the center of gravity according to Equation (3);7. Transform the points in the point cloud *Q* according to Equation (4);8. Minimize the Euler distance between all matches by iteratively using Equation (5);
9. **Return** *R*_1_, *t*_1_.

### 2.5. Pose Correction Node Design

Due to the inevitable errors in interframe pose estimation, the pose correction node was designed. In this node, the NDT algorithm based on dynamic voxel storage is utilized to periodically correct the localization results at a frequency lower than that of the pose estimation node. Specifically, a simple but effective keyframe selection scheme that selects a keyframe every five frames is designed. When a keyframe is selected, the dynamic voxel storage-based NDT algorithm is used to match the keyframe and the local map, and thus localization results of the pose estimation node can be corrected. By combining the pose estimation node and the pose correction node, both the accuracy of the pose estimation of the lidar odometry and the real-time performance of the pose estimation can be guaranteed. The specific algorithm flow for the pose correction node is as Algorithm 2.

First, the target point cloud is divided into grids of a specified size, and the mean vector and covariance matrix of each point in the grids are calculated. Taking one of the grids *y_k_* (*k* = 1, 2, …, *m*) as an example, the calculation formulas are as follows:

(6) $$\left\{\begin{array}{c} \mu =\frac{1}{m}\sum_{k=1}^{m}y_{k}\\ \sigma =\frac{1}{m-1}\sum_{k=1}^{m}(y_{k}-\mu ){\left(y_{k}-\mu \right)}^{T} \end{array}\right..$$

Second, the source point cloud is mapped to each grid through the initial transformation, and the corresponding probability density is calculated according to the mapped grid of the source point:

(7) $$f(x)=\frac{1}{{\left(2\pi \right)}^{D/2}\sqrt{\left|\sigma \right|}}e^{-\frac{{\left(x-\mu \right)}^{T}\sigma^{-2}(x-\mu )}{2}}.$$

In the above formula, *D* represents the *D*-dimensional normal distribution. Then, the dynamic grid map is constructed, and these grids are stored in a map data structure. When the number of points in the grids is greater than the threshold *ɕ*, this grid is considered to be in an active state; only the grids in an active state are used in the following calculation.

When *n* points *q_i_* (*i* = 1, 2, …, *n*) are inserted into a grid containing *m* points with a mean of *μ*, the mean and covariance matrix of each grid can be updated using Formula (8):

(8) $$\begin{array}{l} \mu \leftarrow \left(m\cdot \mu +\sum_{i=1}^{n}q_{i}\right)\cdot \frac{1}{n+m},m\leftarrow n+m\\ \sigma =\frac{1}{m}\sum_{i}(q_{i}-\mu )(q_{i}-\mu {)}^{T} \end{array}.$$

It is clear that Formula (8) can save computational costs and increase speed. In the operation of the algorithm, the dynamic grid map removes the grid outside the radius of the local map, which ensures the dynamic update of the map.

The ultimate purpose of the NDT registration is to find the optimal transformation matrix that enables the point cloud of two frames to achieve optimal matching. The parameters to be optimized are the rotation matrix and translation vector. The parameter *T* = {*R*, *t*} is used to describe the pose transformation, and *Trans*(*T*, *P_k_*) represents the pose transformation of point cloud *P_k_* with the parameter *T*. Then, the likelihood function is constructed as follows:

(9) $$\Theta =\Pi_{k=1}^{n}f(Trans(T,P_{k})).$$

The optimal transformation parameter is *T*, which maximizes the above equation. Maximizing Formula (9) is equivalent to minimizing Formula (10):

(10) $$-\log\Theta =-\sum_{k=1}^{n}\log(f(Trans(T,P_{k})))$$

Then, the Newton iteration method is used to solve Formula (10) iteratively, and the optimal transformation parameter *T* is obtained.
**Algorithm 2:** The pose correction algorithm.**Input:** Point cloud *P*, *Q*, Keyframe selection value *h*, Threshold *ɕ.***Output:** Pose correction result *R*, *t*.1. Determine the keyframe by judging if *h* is equal to five;2. Divide the target point cloud *Q* into grids of a specified size and calculate the mean vector and covariance matrix of each point in the grids according to Equation (6);3. Map the source point cloud *P* to each grid and calculate the corresponding probability density according to the mapped grid of the source point;4. Construct the dynamic grid map and store these grids in a map data structure;5. Determine the activation state of the grids by judging whether the number of points in the grid is greater than the threshold *ɕ*;6. Update the mean and covariance matrix of each grid according to Equation (8);7. Construct the likelihood function according to Equations (9) and (10);8. Use the Newton iteration method to get the optimal transformation parameter;9. Correct the localization results of the pose estimation node;10. **Return** *R*, *t*.

### 2.6. The Overall Framework of the Proposed Localization and Mapping Algorithm

The proposed lidar, camera, and IMU fusion SLAM system includes two main parts: the visual-inertial subsystem and the lidar-inertial subsystem. The visual-inertial subsystem was developed based on the VINS-Mono algorithm, which mainly consists of four threads: the sensor data processing thread, visual-inertial sensor initialization thread, local pose optimization thread, and global pose optimization thread. The lidar-inertial subsystem is based on the LIO-SAM algorithm, which consists of four parts: point cloud projection, feature extraction, and IMU pre-integration and map optimization. In this study, the front end of lidar SLAM was improved, given the characteristics of open space and bumpiness that may exist in the field environments, and the method of extracting the corner and planar points of the point cloud in LIO-SAM was not continued. A joint lidar front end of pose estimation and correction was designed using the Super4PCS, ICP, and NDT algorithms and their variants. The improved lidar odometry can balance localization accuracy and real-time performance by carrying out lower-frequency pose correction based on higher-frequency pose estimation.

**Figure 5:**
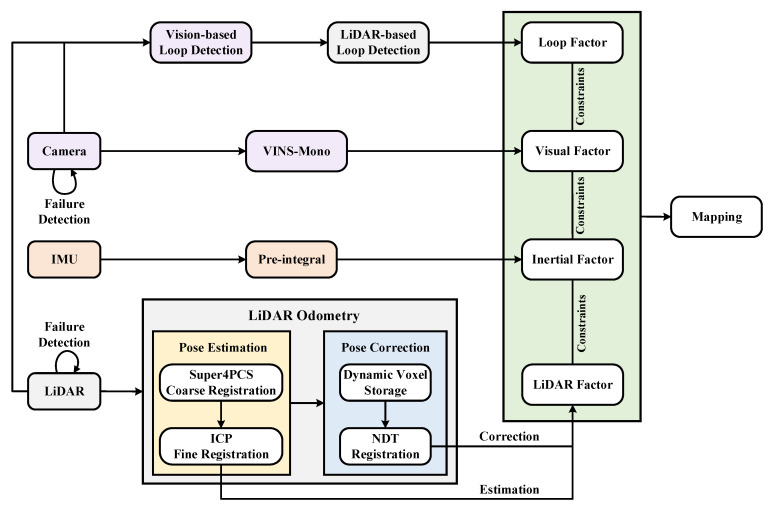
The overall framework of the proposed localization and mapping algorithm.

The overall lidar-vision-inertial SLAM system is a tightly-coupled fusion system, similar to LVI-SAM. The back-end optimization is completed based on the factor graph optimization framework. By establishing the combined residual function of lidar-vision-inertia and conducting nonlinear optimization, the optimal localization and mapping results are obtained. The specific flowchart of the system is shown in Figure 5.

## 3. Results and Discussion

### 3.1. Platform and Experimental Environment

**Figure 6:**
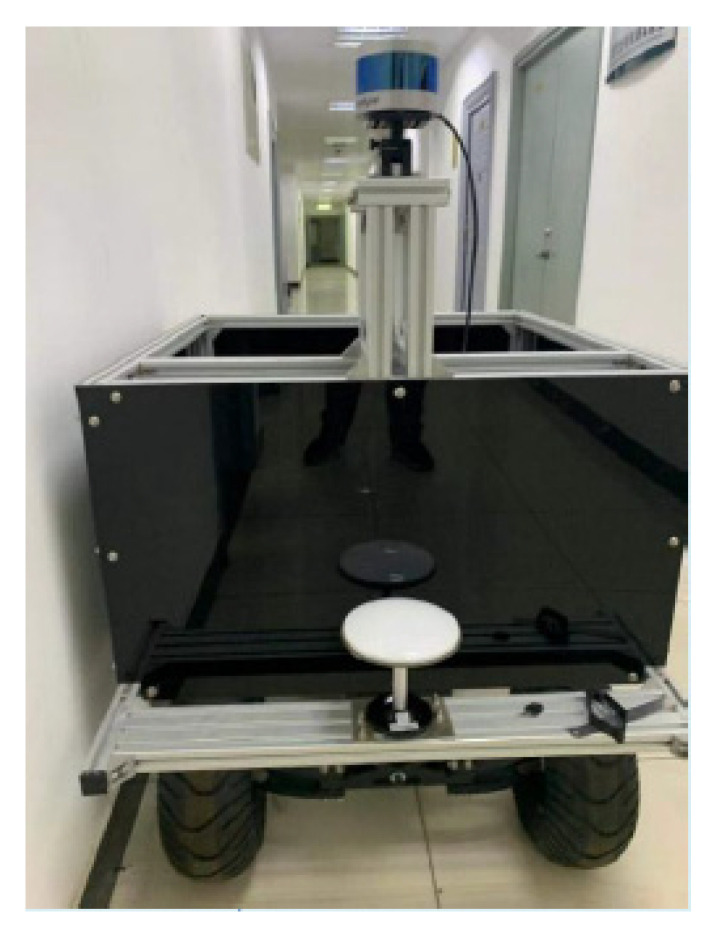
The experimental platform of the proposed localization and mapping algorithm.

A vehicle equipped with various sensors, including a 16-line Lidar, a camera, and a high-precision inertial navigation system, was used as the experimental platform for data acquisition. The speed of this vehicle in the experiment was about 4.8 m/s. The laptop used to run the improved LVI-SAM algorithm was equipped with an Intel i7, 2.20 GHz, 8-core CPU, and 16 GB RAM. The experimental platform is shown in Figure 6 and the specific experimental configurations are shown in Table 1.

The mobile platform used in this study was the HUNTER 2.0. The platform adopts a mature car-like structure, which means that the platform can not only ensure power but also improve the adaptability of the vehicle body to the terrain. For example, it can complete the climbing over 10 cm obstacles and ensure the availability of multiple outdoor scenes, which is very suitable for experiments in the field environment.

The LiDAR sensor module uses the Velodyne VLP-16 LiDAR. The sensor has 16 LiDAR beams for distance measurements, with a measuring distance of up to 100 m, an accuracy of ±3 cm, a vertical viewing angle of ±15°, an angular resolution of 2°, and the working frequency of the module was 5 to 20 Hz. The LiDAR can generate up to 300,000 three-dimensional point cloud data per second and is compact and small enough to be used in the vehicle platforms.

The IMU is the XW-GI5651 inertial navigation unit which outputs high-precision pitch, roll, and yaw angles at an adjustable frequency (1 Hz/5 Hz/10 Hz/100 Hz) (0.1°, 0.1°, and 1°).

The GNSS module, which has a positioning accuracy of less than 2 cm, was used for the acquisition of the ground truth of the pose. The module has two satellite receiving antennas and the real-time kinematic (RTK) technology can be used in network mode to obtain high-precision positioning and orientation information [38].

**Figure 7:**
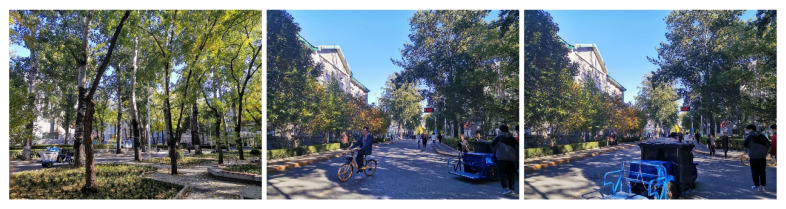
Experimental environments.

To evaluate the effect of the algorithm proposed in this paper, we chose the wild forest and the outdoor road as the data acquisition scenes. The forest is unstructured and bumpy, and there are many dynamic objects on the outdoor road. As the experimental settings showed in Figure 7, there are a great number of structured and unstructured features in the environment, such as vehicles, bicycles, pedestrians, tree lawns, trees, traffic signs, and buildings. All of the above environmental features make it a complicated environment and bring certain challenges to the SLAM algorithm. Therefore, it is suitable to collect data and evaluate the algorithm in the above environments.

### 3.2. Analysis of Localization Results

The absolute pose error (APE) is the error between the estimated pose value and the actual pose value, which can directly reflect the performance of the SLAM system. The APE can be calculated between two poses *P_ref_*_,*k*_, *P_est_*_,*k*_ at timestamp *k*.

(11) $${APE}_{\mathrm{trans},k}=||\mathrm{trans}(P_{ref,k}^{-1}P_{est,k})||$$

(12) $${APE}_{\mathrm{rot},k}=||\mathrm{rot}(P_{ref,k}^{-1}P_{est,k})-I_{3*3}|{|}_{F}.$$

evo (https://github.com/MichaelGrupp/evo (accessed on 20 December 2022.)) is an evaluation tool developed specifically for the SLAM algorithm evaluation. It can easily calculate the APE value of the algorithm, and its evo_res tool can also make an intuitive comparison of the APE values between different algorithms. In this study, APE was used as the evaluation index of localization accuracy.

In the collected dataset, the translation and rotation APE of the proposed algorithm, LVI-SAM, and LIO-SAM were calculated, and the results were compared and analyzed. The comparative results of the localization errors are shown in Table 2.

**Figure 8:**
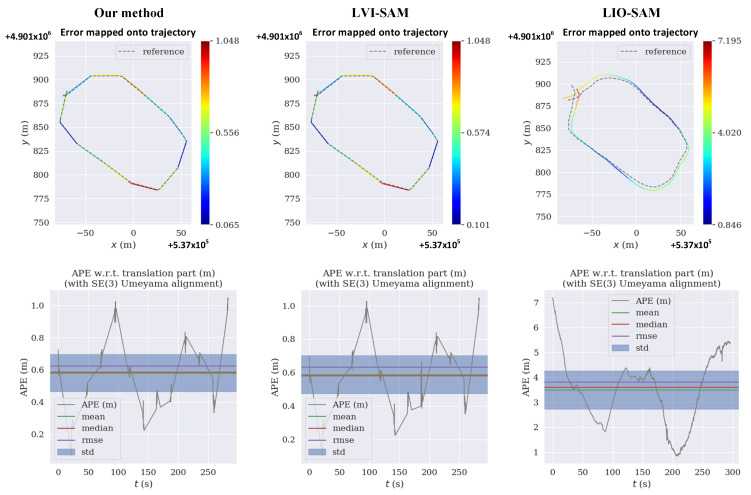
APE of our method, LVI-SAM, and LIO-SAM.

As shown visually in Table 2 and Figure 8, compared with LVI-SAM, the proposed algorithm has a lower the translation error and rotation error, and the root mean squared error (RMSE) index of the translation error was reduced by about 4.7%. Compared with LIO-SAM, the error indexes, and especially the translation error indexes, significantly decreased. The above error indicators suggest that the proposed method is more suitable than the other methods for use in complex field environments.

### 3.3. Analysis of Mapping Results

**Figure 9:**
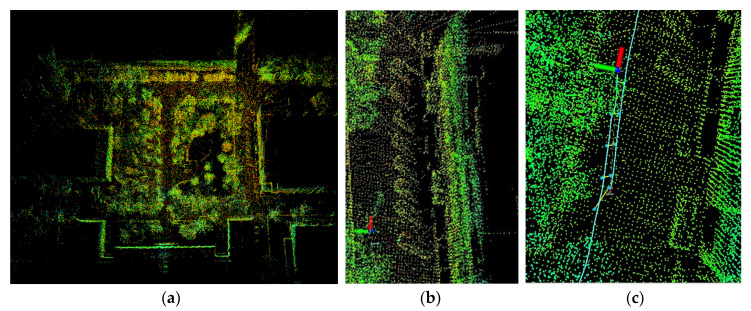
The mapping results of the proposed algorithm in a complex field environment. (**a**) Reconstructed global map of the environment. (**b**) Stationary vehicles in the parking area of the experimental environment. (**c**) Loop closure detection of the proposed algorithm.

Figure 9 shows the mapping results of the proposed algorithm in a complex field environment. Figure 9a is a reconstructed global map of the environment, in which buildings, trees, roads, stationary vehicles, and other objects in the scene can clearly be seen. The established three-dimensional environmental map is highly consistent with the actual scene. Figure 9b shows the stationary vehicles in the parking area of the experimental environment. In the experiment, vehicles pass through the testing site, but no dynamic cars appear in the mapping result, reflecting the accuracy and robustness of the proposed algorithm. In addition, when the closed loop is reached in the real scene, the algorithm also detects the loop closure to complete the global optimization, as shown in Figure 9c.

### 3.4. Analysis of the Real-Time Performance

Implementing real-time point cloud registration in such large-scale field scenarios is challenging. Table 3 shows the average time-consuming results of each point cloud registration step used in the improved SLAM algorithm. It can be summarized from the table that the average time cost for each lidar frame is 354 ms, which can meet the requirements of real-time point cloud registration applications. The time-consuming and the above localization accuracy results jointly suggest that the proposed lidar front end can balance localization accuracy and real-time performance by carrying out lower-frequency pose correction based on higher-frequency pose estimation and the improved SLAM system is suitable for vehicles in complex field environments.

## 4. Conclusions

In this paper, we proposed an improved lidar, inertial, and visual fusion localization and mapping algorithm based on LVI-SAM for vehicles in open, bumpy, and GPS-denied complex field environments. The proposed method optimizes the lidar front end of LVI-SAM and improves the front end based on corner points and planar points in order to combine pose estimation and the correction lidar front end, utilizing the Super4PCS, ICP, and NDT registration algorithms to achieve a balance between localization accuracy and real-time performance. The proposed algorithm was tested in a complex field environment, and the experimental results indicate that the improved LVI-SAM algorithm has high accuracy, high real-time performance, and strong robustness, and can be used for the high-precision localization and mapping of vehicles in complex field environments. The method is of great significance for achieving efficient and stable autonomous driving and judging the surroundings of vehicles in complex field environments.

However, the proposed method still has some limitations. The effectiveness of the proposed algorithm was evaluated on the road of the wild forest environment, which does not include all field environments. Future works include more field area tests including off-road, agricultural, and grassland environments, and the fusion of radar data to the current algorithm in order to cope with weather changes in the field environments.

## Figures and Tables

**Table 1:** Specific experimental configurations.

Platform	Configuration
Vehicle platform	HUNTER 2.0
LiDAR	Velodyne VLP-16
Inertial navigation system	XW-GI5651
Camera	USB 3.1 HD camera
Operation system	Ubuntu 18.04
Software platform	Robot Operating System (ROS) Melodic
Development language	C++
Point cloud processing library	Point Cloud Library (PCL) 1.7
Evaluation tool	evo
Visualization	Cloud Compare

**Table 2:** Comparative results of localization errors in the collected dataset.

	APE	Translation Error (m)				Rotation Error (Unit-Less)			
Algorithm		Max	Mean	Min	RMSE	Max	Mean	Min	RMSE
Our method		1.04	0.58	0.06	0.61	2.82	2.03	0.54	2.16
LVI-SAM		1.05	0.60	0.11	0.64	2.82	2.05	0.59	2.16
LIO-SAM		7.19	3.50	0.85	3.82	2.82	2.82	2.77	2.82

**Table 3:** Time-consuming results of the steps in the improved SLAM system in the collected dataset.

Step	Time Cost (ms)
Point cloud coarse registration	115
Point cloud fine registration	71
Pose correction	168

## Data Availability

The data presented in this study are available on request from the corresponding author.
